# A combination of parabolic and grid slope interpolation for 2D tissue displacement estimations

**DOI:** 10.1007/s11517-016-1593-7

**Published:** 2016-11-11

**Authors:** John Albinsson, Åsa Rydén Ahlgren, Tomas Jansson, Magnus Cinthio

**Affiliations:** 1 Department of Biomedical Engineering, Faculty of Engineering, Lund University, Ole Römers väg 3, 221 00 Lund, Sweden; 2 Department of Medical Imaging and Physiology, Skåne University Hospital, Lund University, Malmö, Sweden; 3 Clinical Sciences Lund, Biomedical Engineering, Lund University, Lund, Sweden; 4 Medical Services, Skåne University Hospital, Lund, Sweden

**Keywords:** Ultrasound, Sub-sample estimation, Block matching, Speckle tracking, In silico, In vivo

## Abstract

Parabolic sub-sample interpolation for 2D block-matching motion estimation is computationally efficient. However, it is well known that the parabolic interpolation gives a biased motion estimate for displacements greater than |*y.*2| samples (*y* = 0, 1, …). Grid slope sub-sample interpolation is less biased, but it shows large variability for displacements close to *y*.0. We therefore propose to combine these sub-sample methods into one method (GS15PI) using a threshold to determine when to use which method. The proposed method was evaluated on simulated, phantom, and in vivo ultrasound cine loops and was compared to three sub-sample interpolation methods. On average, GS15PI reduced the absolute sub-sample estimation errors in the simulated and phantom cine loops by 14, 8, and 24% compared to sub-sample interpolation of the image, parabolic sub-sample interpolation, and grid slope sub-sample interpolation, respectively. The limited in vivo evaluation of estimations of the longitudinal movement of the common carotid artery using parabolic and grid slope sub-sample interpolation and GS15PI resulted in coefficient of variation (CV) values of 6.9, 7.5, and 6.8%, respectively. The proposed method is computationally efficient and has low bias and variance. The method is another step toward a fast and reliable method for clinical investigations of longitudinal movement of the arterial wall.

## Introduction

Tissue motion measurements using ultrasound can provide functional information about the tissue of interest and have attracted attention for various applications such as the evaluation of cardiac [14, 21, 22, 27, 32], vascular [4, 5, 19, 28], and skeletal muscle [9, 18, 30] function.

**Fig. 1:**
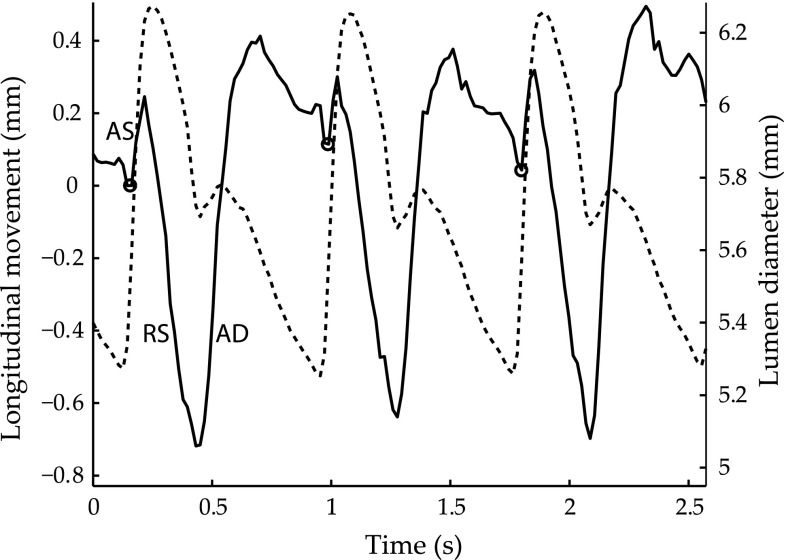
Longitudinal movement (*solid line*) of the intima–media complex of the far wall and the corresponding diameter change (*dashed line*) in the common carotid artery of a 29-year-old female during three cardiac cycles. For longitudinal movement, a positive deflection denotes movement in the direction of blood flow. The *small circles mark* the onset of an antegrade movement in early systole (AS). The distinct antegrade movement is followed by a distinct retrograde movement in systole (RS) and a second distinct antegrade movement in early diastole (AD)

One vascular application of interest is the measurement of the longitudinal movement of the arterial wall, i.e., the motion along the arteries [12, 17, 35, 36]. In large arteries, the displacement is greatest in the layers closest to the lumen—the intima–media complex—and is of the same magnitude as the diameter change [13] (Fig. 1). The outer layer—the adventitia—shows the same basic pattern of movement, but the displacement is smaller, thereby demonstrating the presence of previously unknown substantial shear strain and thus shear stress, intramurally [13, 23, 33, 46]. Recent studies have reported that the amplitude of the longitudinal displacement of the arterial wall is reduced in patients with carotid plaques, suspected coronary artery disease, type 2 diabetes [38–40, 42, 45], and periodontal disease [47], suggesting that the longitudinal movement of the arterial wall might prove to be a valuable marker for future risk of cardiovascular disease. Furthermore, in a study on the porcine carotid artery, we recently reported that longitudinal movement and intramural shear strain undergo profound changes in response to the important endogenous hormones adrenalin and noradrenalin [2]. These findings might have important implications for vascular disease both in the short- and long term and might constitute a link between mental stress and cardiovascular disease [2].

In order to calculate high-resolution motion estimates using block matching, three components are needed. (1) A search method is needed to determine which blocks the kernel should be compared to in order to find the most similar block [24]. (2) A method is needed to determine similarity [20] by calculating evaluation metric values between the kernel and the compared blocks. The value can be either the maximum likeness, e.g., the normalized cross-correlation [37], or the minimum difference, e.g., the sum of the absolute difference [7]. (3) A sub-sample estimation method is needed to determine movements at sub-sample accuracy. The sub-sample estimation method can be one of the following three subgroups: (a) interpolation of the data points [31], (b) interpolation of evaluation metric values [10], or (c) analytically solving the min/max problem for the evaluation metric values [10]. The number of calculations needed for the three subgroups show that for the average method of each group the computational load is highest with (a) and lowest with (c). The motion estimations can be conducted with the ultrasound data in any of several forms, e.g., B-mode, radio-frequency, or after a Fourier transformation.

During the last decade, several tracking methods based on block matching have been developed to measure longitudinal movement of the arterial wall in ultrasound cine loops [3, 12, 13, 15, 34, 41, 44, 45]. The most common method to obtain sub-sample estimations is the use of image interpolation. Interpolation of the image gives good sub-sample estimations but is very time-consuming. Albinsson et al. [3] fitted three evaluation metric values with a parabolic function, which is a computationally efficient method to determine sub-sample displacements. However, it is well known that the parabolic function gives a biased estimation for displacements greater than |*y*.2| samples (*y* = 0, 1, …) [8]. Another sub-sample method, grid slope interpolation [16], gives fast unbiased motion estimates, but it has a large variability of the motion estimates for displacements close to *y*.0 samples (see also below). Considering that the drawbacks of the two sub-sample methods occur at different sub-sample displacements, a possible solution is to combine the two sub-sample methods. We therefore propose a new method, from now on denoted GS15PI, in which the sub-sample displacement is first estimated with a parabolic function. If the absolute sub-sample estimation is greater than a threshold (chosen to be 0.15), the sub-sample estimate is recalculated by grid slope interpolation.

The aim of this work was to evaluate the new sub-sample estimation method and to compare its performance to three sub-sample estimation methods: sub-sample interpolation of the image, parabolic sub-sample interpolation, and grid slope sub-sample interpolation. The evaluations were conducted on simulated and phantom ultrasound cine loops consisting of both B-mode data and radio-frequency data using different settings for the signal-to-noise ratio, velocity, and kernel size. Also, data from an in vivo study of the longitudinal displacement of the common carotid artery in healthy humans were used to evaluate the methods.

## Materials and methods

Ultrasound is a modality based on reflected acoustical waves. The detected oscillating signals are beamformed and saved as radio-frequency (RF) data. A brightness mode (B-mode) image is created from the RF data by envelope detection and scan conversion. In the conversion into B-mode data, the RF data are normally down-sampled in the axial direction and displayed on a logarithmic scale. Thus, the two data types will typically have the same lateral sample distance, whereas the RF data will have shorter axial sample distance. The data points in RF signals are typically called “samples” because they are sampled from the acoustical waves, and the B-mode data points are called “pixels” because they represent the intensity data in an image.

Throughout this text, the word “sample” should be read as “sample and/or pixel” because the effects described are the same for both RF and B-mode data.

### Ultrasound cine loops

Ultrasound cine loops of three types of objects were used: a simulated object, a phantom object, and the far wall of the common carotid artery in vivo.

Ultrasound simulations were created using Field II [25, 26] running under MATLAB R2013a (The MathWorks, Inc., Natick, MA, USA). The settings used in the simulations are presented in Table 1. The in silico model consisted of a body of scatterers with random distribution and scatter power that was displaced a set distance between two images. The cine loops were divided into three groups according to the direction of the displacement: horizontal, vertical, or diagonal (45°). The movement of the scatterers was (0.1; 0.3; 0.5; 0.7; 0.9; 1.2; 1.6; 2.0; 2.4; 2.8) pixels per image in all three groups. From each simulation, three cine loops were created with different levels of signal-to-noise ratio (SNR) by adding white noise to the RF data: no noise, SNR 21 dB, and SNR 16 dB. The RF data were down-sampled by a factor of 16 in the vertical direction during the scan conversion into B-mode data. The settings allowed a pixel density in the B-mode images of 8.1 pixel/mm axially and 4.1 pixel/mm laterally. Motion estimations were conducted using both the RF data and the B-mode data.

**Table 1:** Settings in Field II for the in silico cine loops

Width of element	0.215 mm
Height of element	6 mm
Distance between elements	0.030 mm
Number of elements in transmit/receive	64
Focus on transmission (fixed focal point)	40 mm
Focus on receiving	Dynamic focusing
Elevational focus (acoustic lens)	18 mm
Center frequency	6 MHz
Simulated transducer	LA523 (Esaote SpA, Florence, Italy)
Speed of sound	1540 m/s
Sampling rate	100 MHz
Number of scan lines	128
Size of phantom (width × height × depth)	40 × 50 × 10 mm^3^
Number of scatterers	20,000

**Fig. 2:**
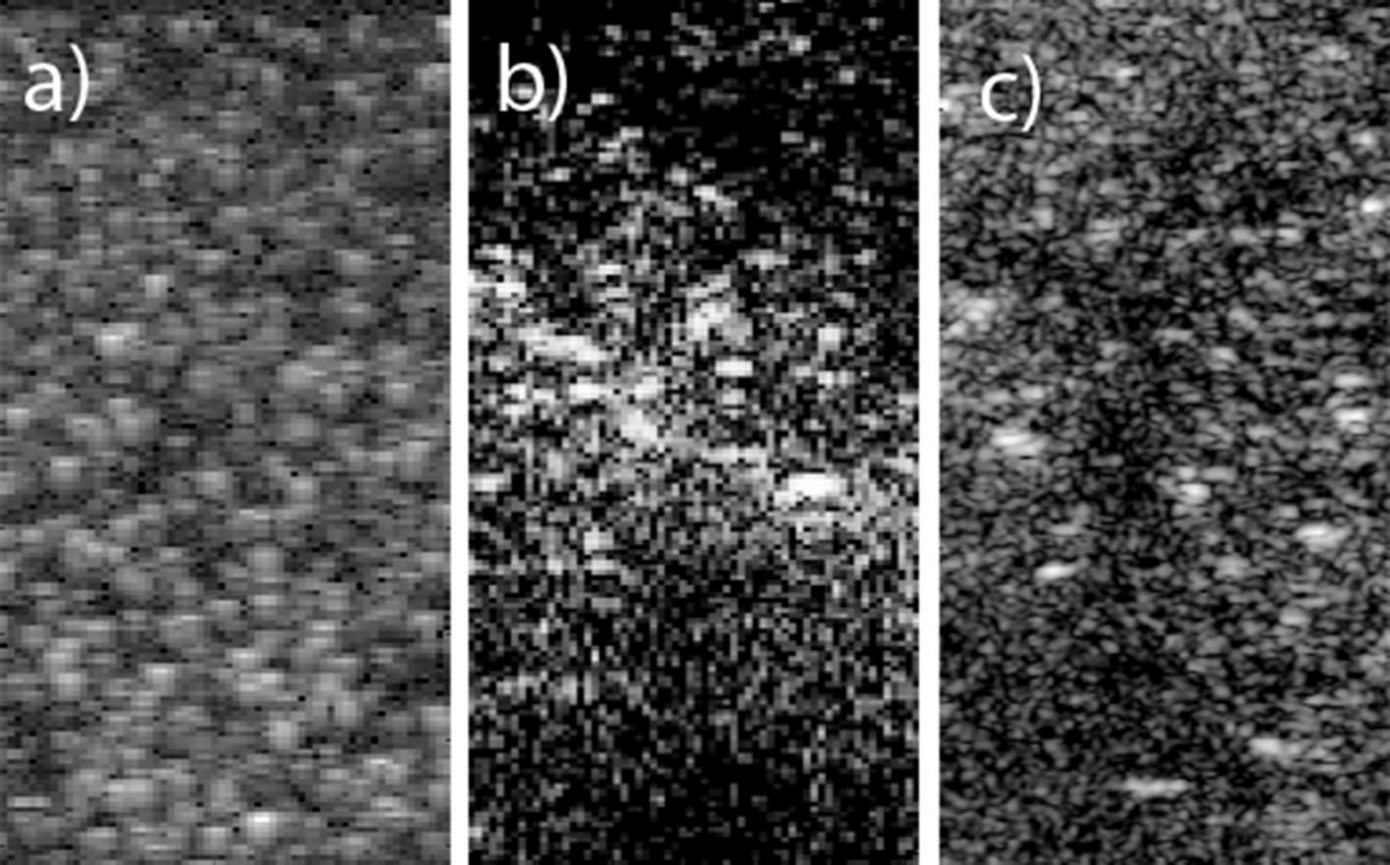
B-mode images from the three ultrasound sources: **a** in silico, **b** ULAOP, and **c** Philips EPIQ 7. The images each depict an area of 20 × 15 mm^2^

Phantom data were collected using both a research ultrasound machine, an Ultrasound Advanced Open Platform (ULAOP) [43] (University of Florence, Italy) equipped with a 4- to 13-MHz linear transducer (LA523, Esaote SpA, Florence, Italy), and a commercial ultrasound machine, a Philips EPIQ 7 equipped with a 3- to 12-MHz linear transducer (Philips Medical Systems, Bothell, WA, USA). Both B-mode data and RF data (down-sampled by a factor of 8 during the scan conversion) were available from ULAOP. The pixel density in the B-mode images was 8.1 pixels/mm axially and 4.1 pixels/mm laterally (the same as the in silico data). Only B-mode data in the DICOM format were available from the Philips EPIQ 7. The pixel density was 21.5 pixels/mm both axially and laterally. Settings were chosen to obtain a frame rate close to 50 Hz using the highest line density, and persistence was turned off in order to avoid averaging between images. The phantom (a sponge) was moved in a water bath at velocities in the range of 2–15 mm/s in steps of 1 mm/s both purely laterally and diagonally within the scan-plane. B-mode data from in silico and phantom measurements are shown in Fig. 2.

The right common carotid artery of 40 healthy volunteers (aged 20–69 years) was examined after at least 10 min of rest in a supine position using a Philips IU 22 equipped with a 5- to 12-MHz linear array transducer (Philips Medical Systems, Bothell, WA, USA). All volunteers gave informed consent according to the Helsinki Declaration, and the study was approved by the Ethics Committee of Lund University. Two cine loops were acquired for each volunteer. Settings were chosen to obtain a frame rate close to 50 Hz using the highest line density, and persistence was turned off in order to avoid averaging between images. DICOM data were exported for off-line motion estimations of the far wall of the common carotid artery. The pixel density was 21.5 pixels/mm both axially and laterally.

### Methods for motion estimation

A tracking scheme for 2D motion estimation was implemented using a full-search method and the sum of the absolute difference as the evaluation metric. The sub-sample positions were determined using four different methods: (1) interpolation of the image data values using cubical splines (CUBIC), (2) parabolic interpolation (PI), (3) modified grid slope interpolation (GSmod), and (4) the proposed method (GS15PI).

#### Search method

The full-search method searched for the best matching block among all possible blocks within a region of interest using the sum of the absolute difference [7] as the evaluation metric. In the B-mode, the size of the region of interest was the size of the kernel + 10 samples both axially and laterally. In the RF data, the size of the region of interest was the size of the kernel + 10 samples laterally, while it was (the size of the kernel + 10) × 16 samples axially. The kernel sizes used in silico and in the phantom measurements were 0.9 × 0.7 mm^2^, 1.8 × 1.7 mm^2^, and 2.8 × 2.7 mm^2^ (Table 2). The kernel sizes for the in vivo motion estimations were visually optimized for each volunteer. The chosen size was used in the two cine loops and for all sub-sample methods.

**Table 2:** Parameters investigated and their different settings

Parameter	Setting
Image data type	B-mode, RF (not Philips EPIQ 7)
Size of kernel: (B-mode in silico and *ULAOP*) [pixels]	7 × 3, 15 × 7, 23 × 11
(B-mode Philips EPIQ 7) [pixels]	17 × 15, 37 × 15, 57 × 55
(RF in silico) [samples]	112 × 3, 240 × 7, 368 × 11
(RF *ULAOP*) [samples]	56 × 3, 120 × 7, 184 × 11
Size of kernel: all cine loops (mm)	0.9 × 0.7, 1.8 × 1.7, 2.8 × 2.7
Noise—SNR	16 dB, 21 dB, no noise
Velocity—direction	Vertical (not phantom), diagonal, horizontal
Velocity—in silico (pixels/frame)	0.1, 0.3, 0.5, 0.7, 0.9, 1.2, 1.6, 2.0, 2.4, 2.8
Velocity—phantom (mm/s)	2, 3, 4, 5, 6, 7, 8, 9, 10, 11, 12, 13, 14, 15

#### Sub-sample estimation methods

CUBIC was used to interpolate the ultrasound data 128 times both axially and laterally using cubical splines [29]. Only the data in the square of the current image centered on the position of the center of the block with the best similarity to the kernel were interpolated. The size of the square was two samples larger than the kernel both axially and laterally. The kernel was not interpolated but was compared to an equal number of interpolated samples obtained at every 128th sample of the interpolated segment. A full search was conducted in the entire interpolated square in order to find the best match at sub-sample resolution.

PI was used to estimate the sub-sample position by fitting a one-dimensional second-degree polynomial to three adjacent evaluation metric values [10] where the center value corresponded to the center position of the block with the best similarity to the kernel. The polynomial was fitted separately laterally and axially. The analytical solution of the polynomial gave the sub-sample estimation as:

1 $$\Delta x = \frac{{\alpha_{1} - \alpha_{3} }}{{2\left( {\alpha_{1} + \alpha_{3} - 2\alpha_{2} } \right)}}$$

where *α*_2_ (center), *α*_1_, and *α*_3_ (on each side of center) denote evaluation metric values and ∆*x* denotes the sub-sample part of the movement.

Grid slope interpolation [16] was used to estimate the sub-sample position by using four evaluation metric values that were calculated between the kernel and four blocks. Two blocks were from the current image—the block with the best similarity to the kernel and the one with the second best similarity. The other two blocks originated in the previous image at the position of the blocks used for the evaluation metric value in the current image. The sub-sample estimation was calculated by:

2 $$\Delta x = 0.5\left( {1 - \frac{{\alpha_{2} - \alpha_{i} }}{{\alpha_{2,0} - \alpha_{i,0} }}} \right)$$

where *α*_2_ (center) and *α*_*i*_ denote evaluation metric values in the current image, and *α*_2,0_ and *α*_*i*,0_ denote the evaluation metric values in the previous image. This method was, in the original work, evaluated on B-mode data using the sum of the absolute difference on horizontal motion only [16].

To expand the utility of the grid slope interpolation methodology, we modified it by setting the variable *α*_2,0_ to zero and setting the variable *α*_*i*,0_ to the evaluation metric value calculated between the best and second best matching blocks in the current image. This resulted in a method denoted GSmod. The sub-sample distance was estimated separately laterally and axially.

Our proposed method, GS15PI, was developed to take advantage of the best characteristics of PI and GSmod. GS15PI first estimates a sub-sample displacement using PI. If the estimated absolute sub-sample displacement is larger than 0.15 samples, the sub-sample estimation is recalculated using GSmod and accepted without further testing. The threshold of 0.15 samples was chosen after an empirical study on phantom movements (unpublished data).

### Evaluation of the motion estimations

The settings that were investigated (summarized in Table 2) covered four sub-sample interpolation methods and five parameters—image data type, kernel size, noise (only for simulated data), direction of movement, and velocity of the object. In this work, two types of results were collected for 100 kernels for each combination of settings—the estimated displacements and the total estimation time used for the sub-sample estimations. Using in silico data, the different combinations noise levels, motion directions, and velocities resulted in 90 cine loops. Using three kernel sizes on each cine loop resulted in a total of 270 parameter settings to be evaluated for each combination of sub-sample method and image type. Using phantom data, there were 84 parameter settings.

A motion estimation error (per image) was defined as the geometrical difference between the set displacement and the estimated displacement, except in Table 4 where the lateral and axial components of the estimation errors were calculated separately. The mean value and standard deviation (SD) were estimated for each setting. The calculation time for a sub-sample motion estimation was measured separately from the search method. The time measurement was taken for 100 sub-sample motion estimations and averaged to give the mean time used for one sub-sample estimation.

The longitudinal movement of the common carotid artery in healthy humans at rest can show dramatically different multi-phasic patterns, even in subjects of similar age and gender [1, 13, 44]. An antegrade longitudinal movement in early systole is followed by a retrograde movement in systole (Fig. 1). The retrograde movement is the most distinct phase, present in all subjects, and is often the largest movement [1, 13, 44]. Therefore, we have chosen to use the magnitude of the retrograde movement in systole when comparing the PI, GSmod, and GS15PI sub-sample estimation methods in vivo. However, in some subjects, the antegrade movement in early systole is absent or very small, which makes the onset of the retrograde movement indistinct. In the present study, these subjects were excluded because the focus of this study was to evaluate the performance of the sub-sample estimation methods and not to evaluate the measurement of the phenomenon itself. Because the magnitude of the longitudinal displacement of the common carotid artery wall seems to decrease with distance from the heart [48], care was taken to perform the measurement at the same position in the two cine loops. The magnitude of the retrograde movement in systole was estimated over the course of 3–5 cardiac cycles per cine loop using a semiautomated method applied to the longitudinal movement curve (Fig. 1). The semiautomated method was initiated by a click on the position of the onset of the antegrade movement in systole. The evaluation of the sub-sample methods was performed by calculating the coefficient of variation (CV) [6] between the mean estimations of the magnitude of the retrograde movement in systole from two cine loops from the same volunteer.

## Results

Using the combined in silico and phantom data, the mean estimation errors were smaller using GS15PI as compared to the other sub-sample methods. GS15PI on average reduced the estimation errors by 14% compared to CUBIC, by 8% compared to PI, and by 24% compared to GSmod. GS15PI also reduced the standard deviations by 12% compared to CUBIC, by 28% compared to PI, and by 2% compared to GSmod. However, there was a large variation in the results depending on the image source and data type in which the motion estimations were conducted (Table 3). In Table 3, the motion estimation errors were calculated using all kernel sizes, motion directions, speeds, and noise levels (where applicable).

**Table 3:** Mean estimation errors and corresponding standard deviation (SD) in µm for sub-sample estimation using in silico and phantom cine loops

Image source	Data type	Sub-sample method			
		CUBIC	PI	GSmod	GS15PI
Philips EPIQ 7	B-mode	11 (8.9)	13 (20)	13 (8.1)	11 (8.5)
ULAOP	B-mode	85 (200)	95 (200)	90 (200)	92 (200)
	RF	78 (190)	77 (200)	67 (200)	72 (200)
In silico	B-mode	41 (140)	49 (140)	69 (140)	48 (140)
	RF	32 (32)	20 (21)	28 (24)	18 (21)

The mean values were calculated over all settings for each data type and sub-sample method. The results are presented according to image data type (B-mode or RF data) and sub-sample estimation method (CUBIC—image interpolation, PI—parabolic interpolation, GSmod—modified grid slope interpolation, and GS15PI—our proposed method)

**Fig. 3:**
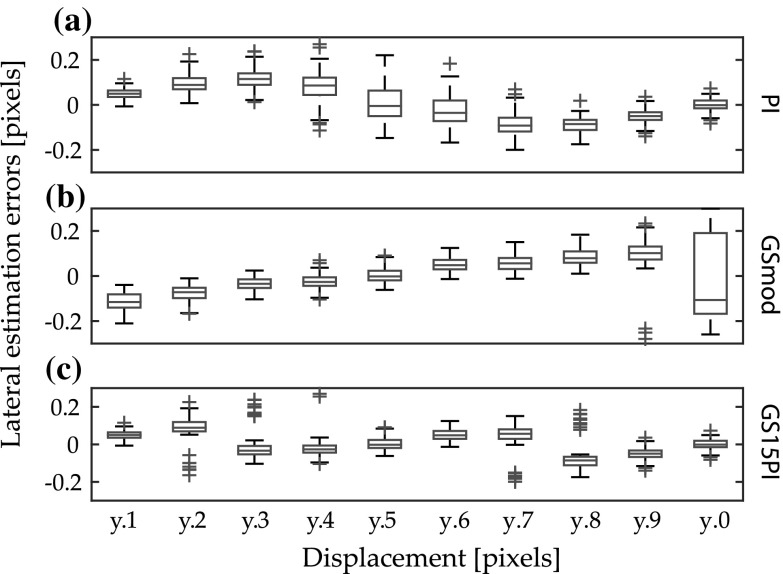
Boxplot of the lateral motion estimation errors for **a** parabolic interpolation, **b** modified grid slope interpolation, and **c** our proposed method in relation to the set sub-sample movement. A total of 100 kernels were used for each velocity. The settings were in silico, B-mode data, horizontal movement, SNR 21 dB, and a kernel size of 1.8 × 1.7 mm^2^. From *left* to *right*, the velocities are 0.1, 1.2, 0.3, 2.4, 0.5, 1.6, 0.7, 2.8, 0.9, and 2.0 pixels/image. The *boxes* indicate the lower and upper quartiles and the median. Outliers are indicated as *crosses*. Bias for each velocity can be seen as a deviation of the median from zero, and variation can be seen by the height of a *box*. Please note that 0.2 pixels ≈ 49 µm

The drawbacks of PI (bias in the motion estimations greater than |*y*.2|) and GSmod (variation in the motion estimations close to *y*.0) were clearly decreased for GS15PI. Figure 3 shows an example of the lateral component of the estimation errors using the following settings: in silico B-mode data, horizontal movement, SNR 21 dB, and a kernel size of 1.8 × 1.7 mm^2^. The bias of PI is visible as a deviation of the median error of each velocity from zero (Fig. 3a). The variation of GSmod is seen as the height of the box for *y*.0 (Fig. 3b). It can also be seen that GSmod had a small linear bias, which is consistent with the result presented by Geiman et al. [16] for the level of SNR in the image data. In Fig. 3c, the improvements in GS15PI can be seen as smaller variation at *y*.0 and median errors closer to zero. However, there are outliers at *y*.2, *y*.3, *y*.7, and *y*.8 (Fig. 3c).

**Fig. 4:**
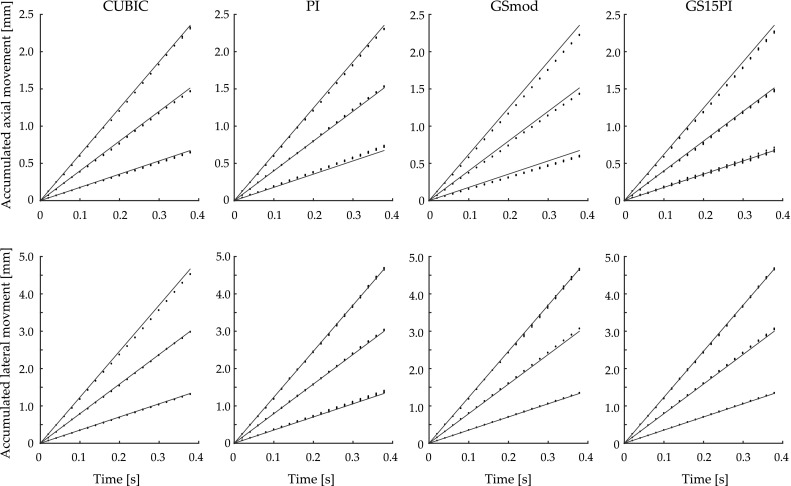
Example of motion estimations in a phantom. The *black lines* indicate the set accumulated displacement for the diagonal motions at 3, 8, and 13 mm/s. The *panels* in the two rows show the accumulated axial and lateral motion estimations of nine kernels using CUBIC—image interpolation, PI—parabolic interpolation, GSmod—modified grid slope interpolation, and GS15PI—our proposed method

The motion estimation errors were analyzed according to axial and lateral estimation errors (Table 4), motion direction (vertical, horizontal, and diagonal) (Table 5), kernel size (Table 6), and noise level (Table 7). As expected, axial errors were smaller than lateral errors, smaller errors were obtained using vertical motion direction than horizontal or diagonal motion directions, and better motion estimations were obtained by larger kernels and less noise. Also as expected, motion estimation using RF data gave smaller errors than using B-mode data, although there were exceptions for the small and medium-sized kernels using data from ULAOP (Table 6). The best results for all sub-sample methods over all image types were obtained using DICOM data from the commercial state-of-the-art Philips EPIQ 7 machine. One exception to the expected results was the larger motion estimation errors using CUBIC on RF data compared to the estimation errors using GS15PI (Tables 3, 4, 5, 6, 7). There was no dependence between the motion estimation errors and the magnitude of the movement. Figure 4 shows an example of estimated accumulated displacements using the four sub-sample interpolation methods in a phantom at three different velocities and diagonal motion direction using the Philips EPIQ 7.

**Table 4:** Mean estimation errors and corresponding standard deviation (SD) values in µm separated according to their axial and lateral components using in silico and phantom cine loops

Image source	Data type	Error component	Sub-sample method			
			CUBIC	PI	GSmod	GS15PI
Philips EPIQ 7	B-mode	Axial	−0.20 (6.0)	0.34 (5.5)	−1.6 (8.1)	0.26 (6.0)
		Lateral	3.5 (12)	−1.1 (23)	2.0 (13)	2.1 (12)
ULAOP	B-mode	Axial	4.3 (99)	4.8 (100)	3.7 (100)	4.8 (100)
		Lateral	0.56 (200)	0.60 (200)	−0.79 (200)	0.88 (200)
	RF	Axial	3.0 (95)	6.7 (95)	6.3 (95)	6.4 (95)
		Lateral	−9.4 (180)	−8.7 (190)	−11 (190)	−8.7 (190)
In silico	B-mode	Axial	15 (62)	19 (62)	24 (62)	17 (62)
		Lateral	35 (130)	41 (130)	60 (130)	41 (130)
	RF	Axial	2.1 (1.8)	0.84 (1.4)	1.3 (1.5)	0.91 (1.4)
		Lateral	32 (33)	20 (21)	28 (24)	17 (21)

The mean values were calculated over all settings. The results are presented according to image data type (B-mode or RF) and sub-sample estimation method (CUBIC—image interpolation, PI—parabolic interpolation, GSmod—modified grid slope interpolation, and GS15PI—our proposed method)

**Table 5:** Mean estimation errors and corresponding standard deviation (SD) values in µm separated according to motion direction using in silico and phantom cine loops

Image source	Data type	Motion direction	Sub-sample method			
			CUBIC	PI	GSmod	GS15PI
Philips EPIQ 7	B-mode	H	4.9 (2.2)	5.5 (4.4)	6.3 (2.0)	4.3 (2.6)
		D	5.6 (3.0)	6.4 (4.2)	6.8 (3.1)	6.2 (3.2)
ULAOP	B-mode	H	13 (8.3)	19 (7.3)	18 (9.3)	16 (8.0)
		D	25 (23)	32 (19)	25 (31)	29 (21)
	RF	H	18 (12)	14 (5.0)	9.3 (5.7)	11 (4.6)
		D	36 (60)	39 (54)	32 (64)	36 (56)
In silico	B-mode	H	2.8 (2.0)	6.0 (2.9)	10 (2.7)	4.9 (2.6)
		V	4.5 (3.4)	6.4 (2.8)	23 (3.5)	6.5 (4.7)
		D	6.2 (4.1)	9.3 (4.7)	11 (4.7)	8.6 (4.5)
	RF	H	12 (9.0)	11 (5.7)	6.8 (5.6)	9.3 (6.1)
		V	5.9 (7.6)	2.5 (1.8)	11 (1.8)	2.5 (1.8)
		D	11 (8.4)	5.1 (2.7)	5.4 (2.0)	3.5 (2.1)

The results are presented according to image data type (B-mode or RF) and sub-sample estimation method (CUBIC—image interpolation, PI—parabolic interpolation, GSmod—modified grid slope interpolation, and GS15PI—our proposed method). The kernel size was 1.8 × 1.7 mm^2^, and for the in silico data, the SNR was 21 dB. Motion direction: V—vertical, D—diagonal, and H—horizontal

**Table 6:** Mean estimation errors and corresponding standard deviation (SD) values in µm separated according to kernel size using in silico and phantom cine loops

Image source	Data type	Kernel size (mm^2^)	Sub-sample method			
			CUBIC	PI	GSmod	GS15PI
Philips EPIQ 7	B-mode	0.9 × 0.8	4.2 (2.4)	5.0 (3.5)	5.0 (2.5)	4.6 (2.6)
		1.8 × 1.7	3.7 (2.0)	4.3 (2.8)	4.6 (2.0)	4.1 (2.2)
		2.8 × 2.7	3.6 (2.0)	4.1 (2.6)	4.5 (1.9)	4.1 (2.1)
ULAOP	B-mode	0.9 × 0.8	74 (85)	75 (85)	76 (87)	75 (85)
		1.8 × 1.7	16 (15)	21 (13)	17 (21)	20 (14)
		2.8 × 2.7	12 (9.4)	18 (4.2)	10 (8.4)	16 (5.0)
	RF	0.9 × 0.8	83 (100)	81 (100)	82 (110)	81 (110)
		1.8 × 1.7	24 (40)	26 (36)	21 (42)	24 (37)
		2.8 × 2.7	12 (8.6)	16 (2.6)	10 (5.7)	13 (4.0)
In silico	B-mode	0.9 × 0.8	43 (67)	45 (65)	46 (68)	44 (67)
		1.8 × 1.7	6.2 (4.1)	9.3 (4.7)	11 (4.7)	8.6 (4.5)
		2.8 × 2.7	4.1 (2.6)	7.4 (3.1)	9.9 (2.3)	6.7 (2.2)
	RF	0.9 × 0.8	14 (11)	7.8 (5.4)	7.8 (5.2)	6.6 (5.2)
		1.8 × 1.7	11 (8.4)	5.1 (2.7)	5.4 (2.0)	3.5 (2.1)
		2.8 × 2.7	8.5 (6.8)	4.4 (1.7)	5.1 (1.3)	2.8 (1.3)

The results are presented according to image data type (B-mode or RF) and sub-sample estimation method (CUBIC—image interpolation, PI—parabolic interpolation, GSmod—modified grid slope interpolation, and GS15PI—our proposed method). The motion direction was diagonal, and for the in silico data, the SNR was 21 dB

**Table 7:** Mean errors and corresponding standard deviation (SD) values in µm separated according to noise level using in silico cine loops

Image type	Noise level	Sub-sample method			
		CUBIC	PI	GSmod	GS15PI
B-mode	SNR 16 dB	9.3 (6.7)	12 (6.1)	16 (6.8)	11 (6.6)
	SNR 21 dB	6.2 (4.1)	9.3 (4.7)	11 (4.7)	8.6 (4.5)
	No noise	5.6 (3.7)	9.1 (4.4)	7.6 (4.2)	7.9 (4.0)
RF	SNR 16 dB	12 (9.0)	4.8 (2.8)	9.6 (2.7)	4.8 (2.6)
	SNR 21 dB	11 (8.4)	5.1 (2.7)	5.4 (2.0)	3.5 (2.1)
	No noise	10 (8.3)	5.3 (2.6)	3.6 (1.6)	3.3 (1.9)

The results are presented according to image data type (B-mode or RF) and sub-sample estimation method (CUBIC—image interpolation, PI—parabolic interpolation, GSmod—modified grid slope interpolation, and GS15PI—our proposed method). The motion direction was diagonal, and the kernel size was 1.8 × 1.7 mm^2^

**Fig. 5:**
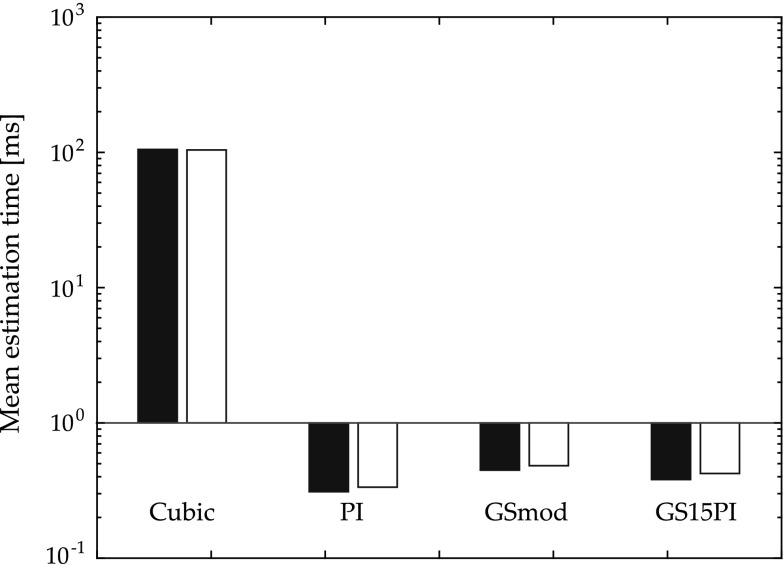
Mean time to perform one estimation using the evaluated sub-sample methods on in silico cine loops for B-mode and RF data. The estimation times are presented according to image data type (B-mode and RF) and sub-sample estimation method (CUBIC—image interpolation, PI—parabolic interpolation, GSmod—modified grid slope interpolation, and GS15PI—our proposed method)

When using in silico data, the mean estimation time was longest for CUBIC followed by GSmod, GS15PI, and PI (Fig. 5). That CUBIC had a more than 27 times longer estimation time than the other methods was expected. When CUBIC was excluded, the sub-sample estimation time was about 1.1 times longer using RF data compared to using B-mode data.

**Fig. 6:**
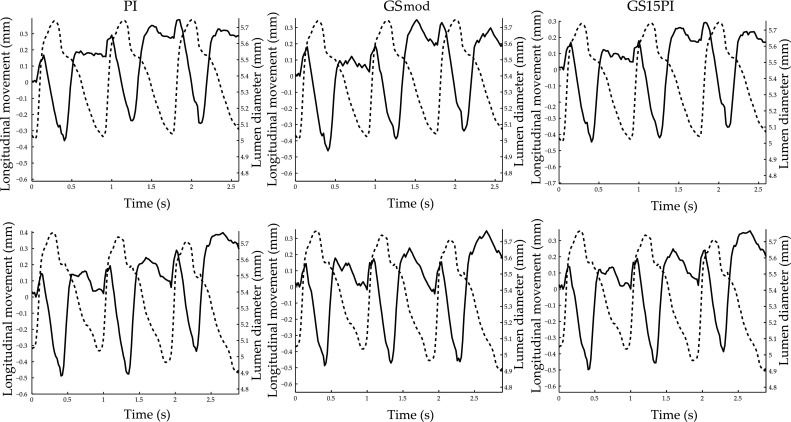
Longitudinal movement (*solid line*) of the intima–media complex of the far wall and the corresponding diameter change (*dashed line*) in the common carotid artery of a 34-year-old female during three cardiac cycles. The *panels* in the two rows show the motion estimations in two different cine loops of the same volunteer using PI—parabolic interpolation, GSmod—modified grid slope interpolation, and GS15PI—our proposed method

The CV values and kernel sizes in vivo were calculated for 21 volunteers (aged 22–67 years) who had a distinct onset of retrograde movement in systole (Fig. 1). The used kernel sizes were in the range of 7 × 13 pixels to 11 × 29 pixels (which is equivalent to 0.29 × 0.55 mm^2^ to 0.46 × 1.22 mm^2^) with a mean kernel size of 7.1 × 25 pixels (0.30 × 1.05 mm^2^). The CV values for the in vivo motion estimations of the retrograde movement in systole using PI, GSmod, and GS15PI were 6.9, 7.5, and 6.8%, respectively. Figure 6 shows in vivo estimations of the longitudinal movement of the common carotid artery wall in one volunteer using the three sub-sample methods.

## Discussion

We have presented a new ultrasound sub-sample motion estimation method, GS15PI, in which the best characteristics of two published methods, parabolic interpolation and grid slope interpolation, are combined to reduce their respective drawbacks of biased and noisy motion estimations (Fig. 3). The performance of GS15PI was evaluated on in silico, phantom, and in vivo cine loops. The new method performed well on both B-mode data and RF data, and the results were at the same level or better for both the magnitude of the motion estimation errors and the estimation time compared to image interpolation and parabolic and grid slope interpolation.

In general, our proposed method showed good stability in its motion estimations. Although the method did not always have the lowest motion estimation errors, the consistently low motion estimation errors resulted in an overall improvement compared to the motion estimation errors of the other sub-sample methods using in silico and phantom cine loops. A decrease in the standard deviation was also noted. This reliability in the motion estimations was also shown in the in vivo study. However, the outliers of the motion estimation errors at *y*.2, *y*.3, *y*.7, and *y*.8 shown in Fig. 3c indicate that the tuning of the method might not be optimal. GS15PI uses a threshold level of 0.15 samples to determine whether to use parabolic interpolation or grid slope interpolation, and a somewhat lower threshold level might have decreased the motion estimation error and variance at *y*.2, *y*.3, *y*.7, and *y*.8. Considering that the threshold was determined using phantom cine loops different from those used in this study (unpublished data), it is possible that the optimal threshold is dependent on some parameter in the cine loops. The implemented threshold was an on/off fixed-value version, and an adaptive version should also be considered in future work.

The results were mostly as expected when analyzing the effects of the different parameters (Tables 4, 5, 6, 7). In line with other studies, the motion estimation errors decreased when the information in the kernel increased with kernel size. It is also known that less noise results in smaller motion estimation errors due to decreased rates of change in the speckle pattern. Different rates of change in the speckle pattern are also the likely cause for the differences in motion estimation errors between the three motion directions. A dependency between the actual distance between the sampled data and the size of the motion estimation errors was observed, i.e., a longer distance between samples resulted in larger motion estimation errors. No dependency between the velocity and the motion estimation errors was seen. This might be due to the limited change in the speckle pattern because we had relatively short movements per frame, and our intention was to have in-plane movements.

The motion estimation errors using CUBIC on RF data were larger than expected when compared to the other sub-sample methods (Tables 3, 4, 5, 6, 7). Because CUBIC performed well using B-mode data, the possibility of an implementation error was unlikely. In order to reduce the computation time during sub-sample estimation, we did not interpolate the kernel. This could be a possible explanation for the increase in these motion estimation errors, and further studies are needed to determine whether this is the case.

A limitation in this study was the absence of tests concerning strain and shearing in the simulations and in the phantom cine loops. Thus, how these motions affect the motion estimation errors of GS15PI compared to the other methods can only be speculated. However, the motion estimation errors using the in vivo cine loops, which incorporate a low level of strain in the investigated area, indicate that GS15PI has some robustness to strain. Further studies are needed to evaluate the influence of higher levels of strain and shearing when using GS15PI. Another limitation in our in vivo evaluation was the exclusion of CUBIC in the motion estimations.

As expected, the computation time for CUBIC was much longer than the other sub-sample methods (approximately 27 times longer) because CUBIC involves many more calculations. This large difference has to be considered when the differences in the motion estimations are small. Please note that the difference in the computation time between the GS15PI and PI or GSmod methods was on the order of 1/10 of a millisecond.

The purpose of the in vivo study in this work was to compare GS15PI to parabolic and grid slope interpolation of in vivo data. Therefore, we wanted a dataset in which the effect of the sub-sample method was dominant and thus we wanted to minimize other sources of error such as the time for the onset of the movement. Therefore, we restricted the comparison to volunteers with a distinct retrograde movement. The in vivo results in this study show promising results with CV values of 7%; however, comparisons with other studies should be made with care. The selection of the volunteers in the present study improves our results compared to other studies, and the improved image quality of modern ultrasound machines likely has an influence on the motion estimations. Previously presented CV values of measurements of the retrograde longitudinal movement of the common carotid artery are 12.5% [11], 16% [44], and 13% [3]. Zahnd et al. [45] reported a CV value of 20% for the total longitudinal movement during a cardiac cycle.

The proposed sub-sample interpolation method, GS15PI, has only been evaluated on the longitudinal wall movement of the common carotid artery in vivo. However, the proposed method has potential to be used in all applications where block matching is used. Further studies are needed to evaluate this.

## Conclusion

The proposed sub-sample method GS15PI, in which the best aspects of parabolic interpolation and grid slope interpolation are combined, was found to have promising performance when compared to three other sub-sample methods with in silico and phantom cine loops of both ultrasound B-mode data and RF data. Compared to parabolic and grid slope interpolation, the proposed method also performed well when estimating the longitudinal movement in the common carotid artery in vivo. The proposed method is computationally efficient compared to image interpolation and has low bias compared to parabolic interpolation and low variance at *y*.0 compared to grid slope interpolation. The method is another step toward fast and reliable clinical investigations of longitudinal movement of the arterial wall.
